# Acariform mites (Acariformes) - permanent symbionts of *Hapalomys delacouri* Thomas (Rodentia, Muridae) in Vietnam

**DOI:** 10.3897/zookeys.459.8952

**Published:** 2014-12-02

**Authors:** Andre V. Bochkov, Alexei V. Abramov

**Affiliations:** 1 Zoological Institute, Russian Academy of Sciences, Universitetskaya Embankment 1, 199034 Saint Petersburg, Russia; 2 Joint Russian-Vietnamese Tropical Research and Technological Centre, Nguyen Van Huyen, Nghia Do, Cau Giay, Hanoi, Vietnam

**Keywords:** Acariformes, Listrophoridae, Myobiidae, *Afrolistrophorus*, *Radfordia*, systematics, rodents, ectoparasites

## Abstract

Two new species of parasitic acariform mites (Acariformes) are described from the Delacour’s marmoset rat *Hapalomys delacouri* Thomas (Rodentia: Muridae) in Vietnam: Afrolistrophorus (Afrolistrophorus) hapalomys **sp. n.** (Listrophoridae) and Radfordia (Radfordia) mirabilis **sp. n.** (Myobiidae). Based on morphological evidences, we show that species of both mite genera associated with *Hapalomys* Blyth do not demonstrate clear phylogenetic links with respective congeners from rodents of the closest genus *Chiropodomys* Peters (Rodentia: Muridae).

## Introduction

Marmoset rats of the genus *Hapalomys* Blyth (Rodentia: Muridae: Murinae) are medium-sized arboreal from Southeast Asia, with highly patchy distributions throughout their range from southern China to the Malay Peninsula. The genus consists of two species, *Hapalomys delacouri* Thomas and *Hapalomys longicaudatus* Blyth (Musser 1972, Musser and Carleton 2005). Little is known about the life history of marmoset rats because of paucity of museum materials available for study. Parasitic mites of marmoset rats have never been reported.

Several specimens of the Delacour’s marmoset rat *Hapalomys delacouri* were collected in southern Vietnam during the mammalogical surveys carried out by the Joint Vietnamese-Russian Tropical Research and Technological Centre (Abramov et al. 2012). In this paper, we describe two new mite species belonging to the families Listrophoridae and Myobiidae (Acariformes) collected from this host. Mites of both families are represented by permanent and highly specialized mono- or stenoxenous ectoparasites inhabiting the fur (Listrophoridae) and skin (Myobiidae) of mammals (Bochkov 2009, 2010).

## Material and methods

In the field, the trapped hosts were individually wrapped in cheesecloth to prevent falling-out of ectoparasites and preserved in 70% ethanol. In the laboratory conditions, mites were collected from ethanol preserved hosts with fine forceps under dissection microscope and mounted in Hoyer’s medium. Specimens were studied using a Leica microscope under phase contrast and Nomarsky (DIC) optics. Drawings were made with a camera lucida, and measurements were taken using a calibrated ocular micrometer. In the descriptions below, the idiosomal setation of listrophorid mites follows Griffiths et al. (1990) with modifications by Norton (1998) concerning coxal setae; the leg setation follows Grandjean (1939a). The idiosomal setation of myobiid mites follows Grandjean (1939b) as interpreted by Bochkov et al. (2008). All measurements are in micrometres (μm), provided for paratypes in parentheses, and were taken as follow: body length = the total length from the anterior extremity of the prescapular shield in listrophorids or the palpal extremites in myobiids to the posterior border of the body; body width = width at the level of setae *se* in listrophorids and setae *c2* in myobiids; length of dorsal shields(listrophorids) = maximum length, measured along the median line of the shields; length of opisthosoma (listrophorids) = length from the posterior margins of trochanter IV insertions to the posterior border of the opisthosoma; length of the posterior legs (listrophorids) = length from the most basal point of the trochanter to the apex of the tarsus, excluding pretarsus; tarsal length was measured without pretarsus. Host systematics follows Musser and Carleton (2005).

Abbreviations of institutions:

UMMZ University of Michigan Museum of Zoology, Ann Arbor, USA;

ZISP Zoological Institute of the Russian Academy of Sciences, St. Petersburg, Russia.

## Systematics

### Family Listrophoridae Megnin & Trouessart Genus *Afrolistrophorus* Fain Subgenus *Afrolistrophorus* Fain

#### Afrolistrophorus hapalomys

Taxon classificationAnimaliaSarcoptiformesListrophoridae

Bochkov & Abramov sp. n.

http://zoobank.org/8AADCBF1-40F9-4A00-9DB6-6ACDEFC2F49B

Figs 1 , 2

##### Type material.

Male holotype (ZISP L-T-9, AVB 10-0803-012), 7 male and 12 female paratypes (ZISP AVB 10-0803-012, 1-19) from *Hapalomys delacouri* Thomas (Rodentia: Muridae) [fur], VIETNAM: Binh Phuoc Province, Bu Gia Map National Park, 13 km NE Bu Gia Map Village, 540 m a.l.s., 12°11'37"N, 107°12'21"E, 13 January 2010, coll. A.V. Abramov (ZISP 99485). Mites removed by A.V. Bochkov.

##### Type deposition.

Holotype and 17 paratypes deposited in ZISP, one female and one male paratypes in UMMZ.

##### Description.

**Figure 1.**
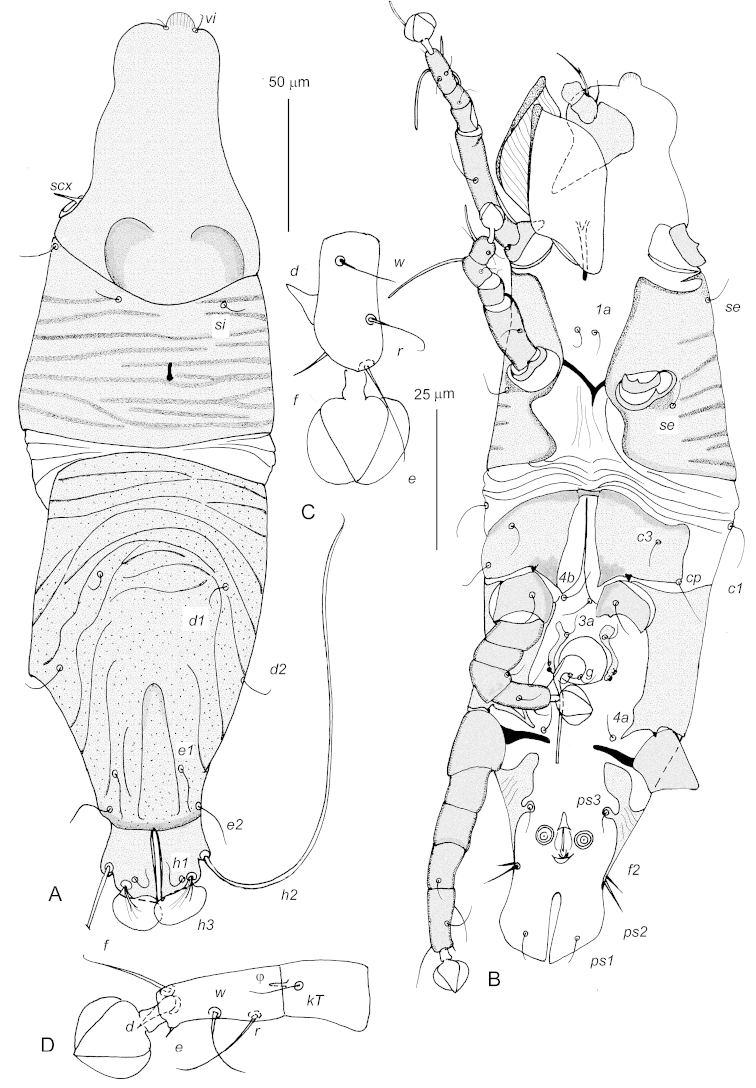
*Afrolistrophorus hapalomys* sp. n., male holotype, **A** dorsal view **B** ventral view **C** tarsus III in ventral view **D** tarsus IV ventral view. Scale bars: **A** and **B** = 50 μm; **C** and **D** = 25 μm.

Male (holotype; paratypes = 7; Fig. 1). Body 360 long (350–385), 105 wide (100–110). Prescapular shield 110 long (105–110) with distinct median process. Postscapular shield 62 long (62-65), covered by 8-10 transverse markings; 2 anterior markings interrupted in median part. Median apodeme present. Hysteronotal shield 155 long (150–160), completely covered by few distinct striae from anterior margin to level of setal bases *e2*; these striae transverse in anterior one third of shield, oblique in middle, and almost longitudinal in posterior one third. Supranal concavity completely sclerotized. Shortest distance between postscapular and hysteronotal shields 10 (10–25). Setae *h2* 155 long (140–160); membranous setae *h3* well developed, about 35 wide, slightly overlapping, without ribs. Terminal cleft 30 long (30–37). Opisthosomal lobes about 20 maximum wide. Cuticle between coxal fields II not striated. Coxal apodemes III fused to each other. Aedeagus about 45 long. Diameter of adanal suckers about 8. Legs III and IV about 75 and 90 long, respectively. Tarsus III without dorso-subapical projection. Tarsi III 20 long (20–23) and tarsi IV 25 long (25–30). All setae of tarsi III and IV shorter than respective segments, excluding pretarsi; setae *d* III and *d* IV spur-like, setae *e* IV microspines. Solenidia *ω1* I, II 12–15 long, *ω3* I about 25 long, *φ* I, II 40–45 long.

**Figure 2.**
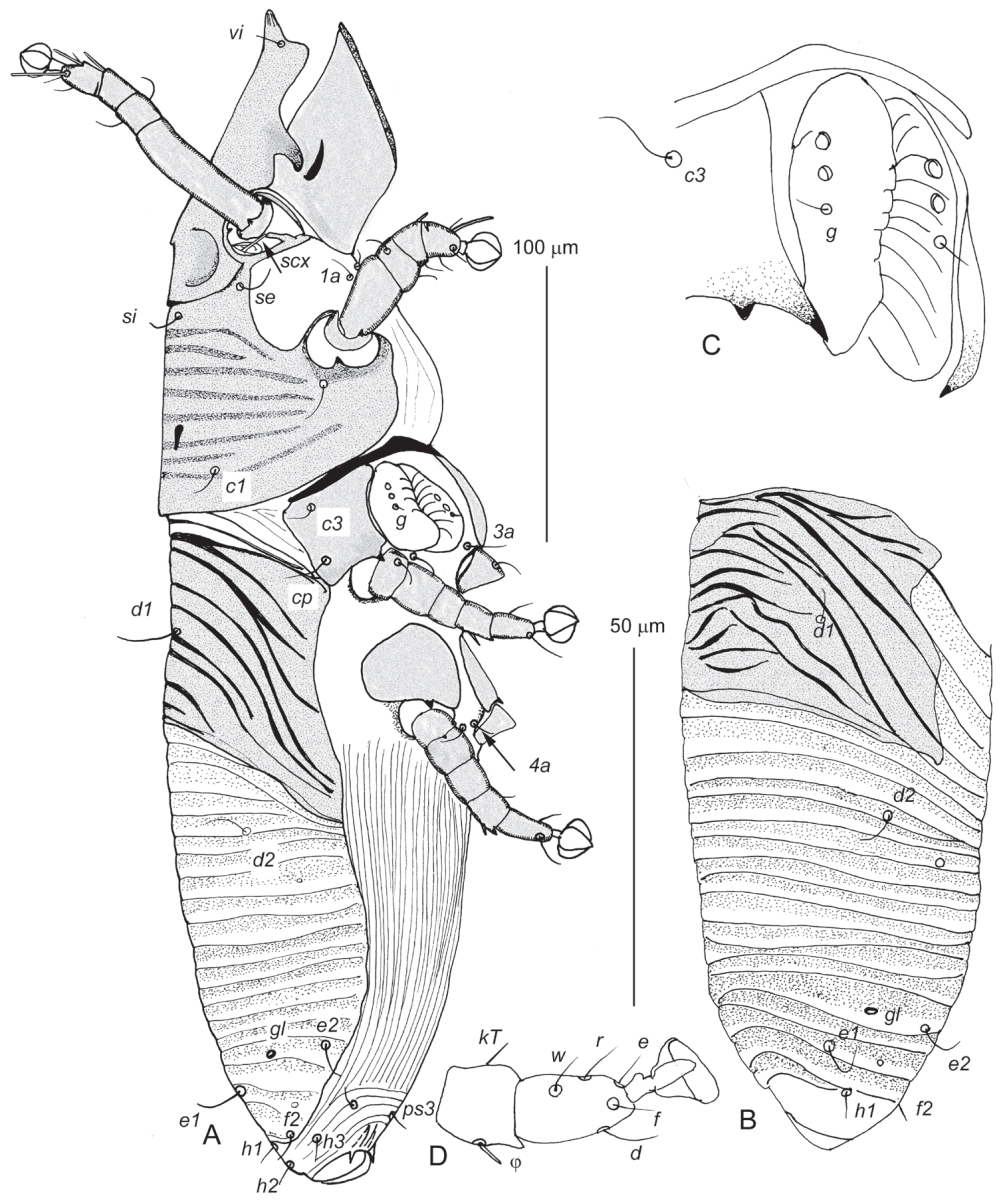
*Afrolistrophorus hapalomys* sp. n., female, **A** lateral view **B** opisthosoma in dorsal view **C** ovipore **D** tibia and tarsus III in ventral view. Scale bars: **A** and **B** = 100 μm; **C** and **D** = 50 μm.

Female (ranges for 10 paratypes, Fig. 2). Body 425–440 long, 100–115 wide. Prescapular shield 110–120 long. Anterior margin of prescapular shield with distinct median process. Postscapular shield about 75 long, covered by 7-9 transverse markings. Median apodeme present. Idiosomal surface between postscapular and hysteronotal shields with 3–4 transverse lines. Hysteronotal shield 70–80 long, crossed by 8–11 oblique striae, 3 posterior striae very short, situated medially. Hysteronotum posterior to hysteronotal shield with 18–20 transverse striae, sclerotized, but less than this shield properly. Opisthosoma about 180 long. Posterior end of opisthonotum without lateral sclerotized patches. Cuticle between coxal fields II not striated. Opisthogaster without scales or verrucae. Setae *h2* short, about 8 long, subequal in length to other opisthosomal setae. Setae *ps1, ps2*, and *4b* absent. Legs III and IV subequal, 65–70 long. Setae *d* III and *d* IV about 2 times shorter than respective tarsi, excluding pretarsus. Solenidia *ω1* I, II about 12 long, *ω3* I about 24 long, *φ* I, II about 8 long.

##### Etymology.

The species name is derived from the generic name of the host and is a noun in apposition.

##### Differential diagnosis.

This new species belongs to the “*apodemi*” species group, which includes twelve species parasitizing mostly Eurasian murines (Murinae). All species in this group have a median process on the anterior margin of the prescapular shield. In males, apodemes III are fused to each other; in females, the cuticle between coxal fields II is without distinct striations, setae *ps1* and *ps2* are either present or absent, setae *h2* are not longer than other opisthosomal setae (Bochkov and OConnor 2006; Bochkov et al. 2011). Among species of the “*apodemi*” group, the new species is close to *Afrolistrophoroides laonastes* Bochkov et al., 2011 from *Laonastes aenigmamus* Jenkins et al. (Rodentia: Diatomyidae) (Bochkov et al. 2011). In both species, the postscapular shield is distinctly developed and possesses several transverse markings and lacks a median sclerotized band; seta *d* III is much shorter than the respective tarsus (excluding the pretarsus); in males, the hysteronotal shield is ornamented in posterior part, setae *h3* are strongly widened and slightly overlap each other; in females, setae *4b*, *ps1* and *ps2* are absent, the ventral side of opisthosoma has no verrucae or scales. *Afrolistrophorus hapalomys* sp. n. differs from *Afrolistrophoroides laonastes* by the following characters. In both sexes of *Afrolistrophorus hapalomys*, the postscapular and hysteronotal shields are covered by a few markings or striae (less than 15), setae *d* IV of tarsi IV are at least twice as short as the respective segment; in males, the supranal concavity is completely sclerotized, tarsi IV are without projections; in females, most striae of the hysteronotal shield are oblique, the posterior end of the opisthonotum is devoid of the lateral sclerotized patches. In both sexes of *Afrolistrophoroides laonastes*, the postscapular and hysteronotal shields are covered by numerous markings or striae (more than 20), setae *d* IV of tarsi IV are subequal or longer than the respective segment; in males, the supranal concavity is not sclerotized, tarsi IV have a distinct subapical projection; in females, striae of the hysteronotal shield are relatively straight, the posterior end of the opisthonotum has a pair of the lateral sclerotized patches.

### Family Myobiidae Megnin Genus *Radfordia* Ewing Subgenus *Radfordia* Ewing

#### Radfordia mirabilis

Taxon classificationAnimaliaTrombidiformesMyobiidae

Bochkov & Abramov sp. n.

http://zoobank.org/0B4528B8-9C9B-4C20-8FF3-558911D7363F

Fig. 3

##### Type material.

Female holotype (ZISP My-T-37, AVB 10-0803-012) and 1 female paratype (ZISP AVB 10-0803-012) from *Hapalomys delacouri* Thomas (Rodentia: Muridae) [skin], VIETNAM: Binh Phuoc Province, Bu Gia Map National Park, 13 km NE Bu Gia Map Village, 540 m a.l.s., 12°11'37"N, 107°12'21"E, 13 January 2010, coll. A.V. Abramov (ZISP 99485). Mites removed by A.V. Bochkov.

##### Type deposition.

Holotype and single paratype deposited in ZISP.

##### Description.

Female (holotype, 1 paratype). Body 435 long (410), 245 wide (230). Body 1.8 times longer than wide. Setae *vi*, *ve*, *d1*, and *d2* 10–12 wide at base; *si* and *se2* about 7 wide at base; *c1* and *c2* about 5 wide at base, *e1*, *e2*, and *f2* about 3 wide at base. Apices of setae *si* reaching level of setal bases *d1*, apices of setae *se* reaching level of setal bases *c2*. Approximate distances between bases of setae: *vi*–*vi* 25, *si*–*si* 22, *c1*–*c1* 17, *c1*–*c2* 50, *d1*–*d1* 15, *d2*–*d2* 50, *c1*–*d1* 52, *d1*–*d2* 17. Setal bases *f1* situated close to *e2* than to *e1*, distance *e1*–*f1* about 50, *f1*–*e2* about 13. Setae *f2* situated at lateral margins of idiosoma. Lengths of setae: *vi* 75 (70), *ve* 90 (93), *si* 115 (110), *se* 87 (85), *c1* 63 (75), *c2* 125 (115) – all distinctly longitudinally striated; *d1* 115 (117) and *d2* about 125, membranous, without striae, *e1* about 27, *e2* about 75, *f1* about 60, *f2* about 15, *h1* about 12, *h2* 310 (315), *ps1* and *ps2* about 11, *ps3* (hook-like) about 15, *g1* about 6, *g2* about 8, *1a, 2a* 25–28, *3a* about 80, and *4a* about 75, *ag1* and *ag3* 8–11, *ag2* about 12. Setae *3a* and *4a* slightly thickened. Apical segment of leg I without ventral projection. Setation for legs II-IV (solenidia in parentheses): tarsi 7(1)-6-6, tibiae 6-6-6, genua 7 (1)-6-5, femora 5-3-3, trochanters 3-3-3, coxae 4-2-2.

##### Etymology.

This epithet refers the unusual external morphology of this species – *mirabilis* (Latin, wonderful).

##### Differential diagnosis.

The subgenus *Radfordia* is separated onto two species groups, “*ensifera*” (setation of coxae II-IV 3-2-2) and “*lancearia*” (3-1-2) (Bochkov and Fain 2003; Bochkov 2009). The new species distinctly differs from all known species of both groups by the setation of coxae II-IV(4-2-2), position of setae *f2* on the lateral margins of the opisthosoma (vs. distant from the lateral margins in all other species) and the bases of *f1* and *e2* which are situated close to each other (vs. distant in all other species). Therefore, we establish for this new species a new species group *mirabilis*.

**Figure 3.**
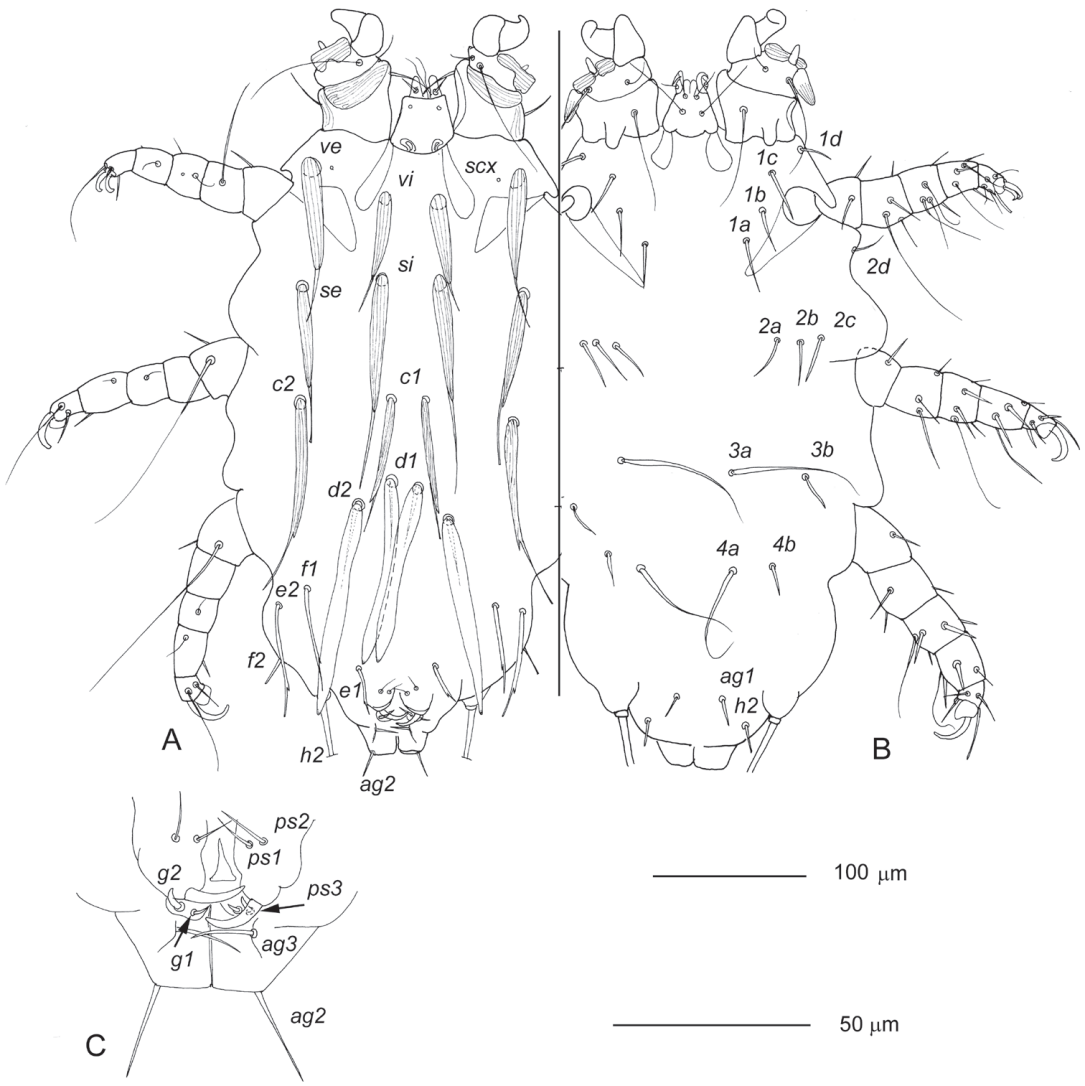
*Radfordia mirabilis* sp. n., female holotype, **A** dorsal view **B** ventral view **C** vulva. Scale bars: **A** and **B** = 100 μm; **C** = 50 μm.

## Discussion

The phylogenetic position of *Hapalomys* is still unclear because of the scarcity of museum specimens. Usually the genus is placed within *Micromys* division of the large muroid subfamily Murinae (Musser and Carleton 2005). Other authors suggested a close link between *Hapalomys* and *Chiropodomys* (Misonne 1969; Musser and Newcomb 1983; Chaimanee 1998; Musser and Carleton 2005). The both mite species from *Hapalomys* described herein strongly differ from the respective congeners described from rodents of the genus *Chiropodomys* Peters (Fain 1970, 1976; Bochkov and Fain 2003). *Afrolistrophorus chiropodomys* Fain, 1970 described from *Chiropodomys major* Thomas and *Afrolistrophorus hapalomys* sp. n. belongs to the same species group “*apodemi*” but in the limits of this group they strongly differ from each other by the ornamentation of the dorsal shields in both sexes. *Radfordia chiropodomys* Fain, 1976 described from *Chiropodomys gliroides* (Blyth) is the typical representative of the species group “*ensifera*” (Bochkov and Fain 2003), whereas *Radfordia mirabilis* sp. n. is a sole representative of a separate species group and morphology strongly different from all other representatives of this subgenus (see description). Based on morphological evidences, we conclude that species of both mite genera associated with *Hapalomys* Blyth do not demonstrate clear phylogenetic links with respective congeners from rodents of the closest genus *Chiropodomys*.

## Supplementary Material

XML Treatment for Afrolistrophorus hapalomys

XML Treatment for Radfordia mirabilis
